# COVID‐19 disparities by gender and income: Evidence from the Philippines

**DOI:** 10.1111/ilr.12226

**Published:** 2022-02-22

**Authors:** Rouselle F. LAVADO, Keiko NOWACKA, David A. RAITZER, Yana van der Meulen RODGERS, Joseph E. ZVEGLICH

**Affiliations:** ^1^ World Health Organization, (at the time of writing, affiliated to the Asian Development Bank); ^2^ Asian Development Bank; ^3^ Rutgers University

**Keywords:** COVID‐19 pandemic, gender gap, employment, unpaid work, poverty, lockdown, women, Philippines

## Abstract

The COVID‐19 pandemic and the resulting containment policies have hit the Philippines harder than most developing countries. The government lockdown is among the strictest in the world, and blanket school closures are the lengthiest. This article uses a novel simulation model to estimate the gendered and regional impacts of these factors on labour, income and poverty, and a case study of school closures points to the losses in employment among private school teachers and in the income of parents with young children. The authors find that the pandemic has had unprecedented implications for economic activity and has disproportionately affected women.

## 1. Introduction: COVID-19 and gender

The COVID-19 pandemic has led to immense losses in health and well-being for individuals, households and societies around the globe. Growing evidence of a gender disparity in the case fatality rate of COVID-19 across countries indicates a disadvantage for men, but the gender differential is reversed for other, non-physiological dimensions of the disease (Bahn, Cohen and Rodgers 2020; Wenham, Smith and Morgan 2020). Of particular concern is the overrepresentation of women among low-wage frontline workers who are at greater risk of exposure to the virus. Women have also experienced relatively greater employment losses

* World Health Organization, email: lavador@who.int (at the time of writing, affiliated to the Asian Development Bank). ** Asian Development Bank, emails: knowacka@adb.org, draitzer@adb.org and jezveglich@adb.org. *** Rutgers University, email: yana.rodgers@rutgers.edu (corresponding author). The authors would like to thank Glenita Amoranto, Liming Chen and Xylee Javier for their helpful contributions to the simulation modelling. They would also like to thank Esther Barrett and Yasuyuki Sawada, and participants in the 2021 Asian Development Bank Economists' Forum for their feedback on an earlier draft. The views expressed in this article are those of the authors and do not necessarily reflect the views and policies of the Asian Development Bank or its Board of Governors, or of the governments they represent.

Responsibility for opinions expressed in signed articles rests solely with their authors, and publication does not constitute an endorsement by the ILO.

This article is also available in French, in *Revue internationale du Travail* 161(1), and Spanish, in *Revista Internacional del Trabajo* 141(1).

[Correction added on 26 March 2022, after first online publication: The copyright line was changed.]

in many countries, given that retail, food services and hospitality are among the most affected industries, and that women are disproportionately represented in them (Alon et al. 2020; ILO 2021). A growing volume of global evidence indicates that the increase in care work during the pandemic has fallen disproportionately on the shoulders of women (Craig and Churchill 2021; İlkkaracan and Memiş 2021; Zamarro and Prados 2021). Evidence has also emerged to indicate that low-income and precariously employed workers and their families are bearing relatively more of the costs of the stay-at-home orders across countries (Kabeer, Razavi and Rodgers 2021). Migrant workers have emerged as a particularly vulnerable group in a diverse range of contexts, their tenuous situation often exacerbated by travel bans, exclusions from emergency public assistance, poor housing, violations of worker rights, and immigration restrictions (Guadagno 2020; Koh 2020; Stevano et al. 2021).

Among developing countries, the Philippines is one of the hardest hit. Global data on COVID-19 infections indicate that the Philippines has more COVID-19 laboratory-confirmed cases and deaths per capita than many other countries in Asia, as well as the highest number of diagnosed cases per capita in Southeast Asia. At the peak of the crisis in April 2021, the Philippines had 1,351 biweekly confirmed cases per million people, which exceeded the world average of 1,215 and the case numbers in every country in Asia except for India.[1] As in the majority of countries, both morbidity and mortality from COVID-19 in the Philippines have affected men more than women: in April 2021, 53 per cent of confirmed COVID-19 cases and 60 per cent of deaths were male, compared with global averages of 54 per cent for COVID-19 cases and 59 per cent for deaths.[2] However, emerging evidence suggests that the gender differential is reversed in the burden imposed by containment policies, especially in terms of women's employment losses, relatively greater unpaid care work burden at home, mental health and domestic violence (UN Women and Women Count 2020; Cho et al. 2021; De los Santos and Labrague 2021).

Like many other Asian governments, the Government of the Philippines imposed a lockdown in mid-March 2020. This lockdown was one of the strictest and longest worldwide (Hapal 2021). It included blanket school closures that were mandated to continue until widespread vaccination of the population had been achieved (which may not be possible for several years), even though children in most other countries had returned to school by late 2020 (Raitzer et al. 2020). The Philippines constitutes an important case study not only because it is one of the hardest-hit developing countries with the longest stringent lockdown, but also because it is seen as a leader on gender equality in the region, with strong gender laws and high rankings for several key indicators of gender equality, including those for wages, education and political participation.

[1] Hannah Ritchie, Edouard Mathieu, Lucas Rodés-Guirao, Cameron Appel, Charlie Giattino, Esteban Ortiz-Ospina, Joe Hasell, et al., "Coronavirus Pandemic (COVID-19)" online database. https://ourworldindata.org/coronavirus (accessed April 2021).

[2] Global Health 50/50, "The COVID-19 Sex-Disaggregated Data Tracker", The Sex, Gender and COVID-19 Project database. https://globalhealth5050.org/the-sex-gender-and-covid-19-project/ (accessed April 2021).

This article explores the impact of COVID-19 in the Philippines by analysing the differential effects of the lockdown by gender, region and income group. To this end, we use a novel simulation model based on the Labor Force Survey (LFS) and the Family Income and Expenditure Survey (FIES) of the Philippines to estimate the impact of the pandemic and lockdown policies on labour, income and poverty, and how these effects differ by gender and region. Through our case study, we also examine how school closures have additional labour market consequences, in terms of losses both in employment of private school teachers and in the income of parents who have had to provide childcare and homeschooling. We find that the COVID-19 crisis and lockdown policy responses have had unprecedented implications for economic activity in the Philippines and have disproportionately affected women. This study should help to inform gender-aware policy responses that put due emphasis on gender equality and poverty reduction.

The remainder of this article is organized as follows. The second section provides a background to the situation in the Philippines. The third section sets out the situational model that we apply to estimate the differential impacts of the pandemic and containment policies, and the fourth section presents our results. The fifth section focuses specifically on the effects of school closures on employment and income, and the sixth section concludes by summarizing our findings and outlining their policy implications.

## 2. Country background

The Philippines is often ranked as one of the top performers in Asia for several key indicators of gender equality, including those for wages, educational attainment and political empowerment. For example, in 2019 the World Economic Forum (WEF) ranked the Philippines 14th out of 153 countries for economic participation and opportunity, and 29th for political empowerment, outperforming regional neighbours with higher levels of income, such as Japan, the Republic of Korea and Singapore (WEF 2019). Reflecting a relatively high level of female political participation, women make up 28 per cent of parliament members, compared with a global average of 25 per cent and a regional average of 20 per cent (UNDP 2020). Possibly in response to the severe lockdown, the Philippines slipped in both WEF rankings in the 2021 report, to 18th and 33rd, respectively (WEF 2021). The Philippines has also achieved gender parity in educational attainment and is one of few low- and middle-income countries in which most cohorts of women have an advantage over men in terms of obtaining a formal education. In addition, a fairly high proportion of female paid employees have a college education compared with other countries at comparable levels of development, and the Philippines is conspicuous for having near gender earnings parity – a rarity globally, not just for its income level (ADB 2015; Zveglich, Rodgers and Laviña 2019).

The 2008 Magna Carta for Women is widely recognized as a landmark legal document, with a mandate to combat discrimination, protect women's human rights, and promote gender equality.[3] These substantial advances are the

[3] Republic Act No. 9710, *Official Gazette*, 14 August 2009.

outcome of proactive policy measures and civil society activism conducted against a backdrop of strong economic growth. Despite these efforts and achievements in terms of wages, politics and education, the Philippines lags behind in other key indicators, which helps to explain its rather lacklustre ranking of 104th in the United Nations Development Programme's Gender Inequality Index (UNDP 2020). In this index, the Philippines does even less well on women's reproductive health status, as reflected by a relatively high maternal mortality rate and adolescent birth rate, and it has a fairly low female labour force participation rate compared with its regional neighbours and even with the global average (UNDP 2020).

The COVID-19 pandemic thus represents an important test of the resilience of progress made to close the gender gap in several key indicators of empowerment, and of its robustness against such an overwhelming socio-economic shock. It also tests the extent to which this progress has fallen even further behind in other areas, especially in terms of women's labour force participation and reproductive health.

Globally, the pandemic is already seen to threaten gender equality gains, with predictions that decades of progress in this domain will be lost. Even though female morbidity due to COVID-19 remains lower than that of men, women and girls are disproportionately negatively affected by containment policies. The ILO has estimated that job losses for women will be greater than for men: 527 million women (or 41 per cent of total female employment) work in hard-hit vulnerable sectors, compared with only 35 per cent of total male employment (ILO 2020).[4] School closures are also predicted to lead to 11 million girls remaining out of school (UNESCO 2020). The closure of day-care centres and schools has increased the amount of time women spend on providing care, reinforcing the unequal intra-household distribution of unpaid care work. In Asia and the Pacific, women spend on average four times more time on unpaid care work than men (ILO 2018). Maternal and infant mortality rates are expected to rise due to disruptions in maternal and child health services, with estimates of 56,700 additional maternal deaths across 118 low- and middle-income countries (Roberton et al. 2020). Rates of intimate-partner violence have also increased, linked to lockdown measures and social and economic stress. Compared with pre-COVID-19 figures, local media and civil society organizations have noted increases across Asia and the Pacific in the volume of domestic violence hotline calls, ranging from 33 per cent to more than double, and in the number of women trying to access dedicated domestic violence shelters (ESCAP 2020).

Patterns in available statistics suggest that the Philippines has also faced these global trends. The Inter-Agency Task Force for the Management of Emerging Infectious Diseases has been announcing its containment measures as levels of "community quarantine", which include industry-specific restrictions on employees permitted at workplaces. Quarantine levels are also spatially differentiated, with the most stringent restrictions applied in the major metropolitan areas where most COVID-19 cases have arisen. The strictest level of quarantine,

[4] These sectors include accommodation and food services; real estate, business and administrative activities; manufacturing; and wholesale/retail trade.

“enhanced community quarantine”, is broadly a stay-at-home order. Under this quarantine level, the population is expected to stay at home except for essential errands (such as purchasing food or medicine) and employees are allowed into workplaces only in essential industries. “General community quarantine”, which was introduced in May 2020, is less strict and allows public transportation at reduced capacity, as well as business operations with reduced numbers of workers and customers. “Modified general community quarantine” further relaxes the restrictions and allows most businesses to operate in areas with few COVID-19 cases.

Published aggregate data based on the Philippines’ LFS indicate that unemployment rose from 5.2 per cent in April 2019 to 17.6 per cent in April 2020, when business closures surged, with men accounting for a larger proportion of the unemployed. Labour force participation dropped from 61.3 to 55.7 per cent during the same period, with women accounting for the majority of those outside the labour force (PSA 2020). Moreover, survey data indicate that Filipino women experienced more adverse psychological effects from the pandemic and higher levels of stress, anxiety and depression (Tee et al. 2020). The pandemic also contributed to problems and disparities in the home. Domestic violence, already a long-standing problem in the Philippines, also increased under the strict lockdown protocols (Sediri et al. 2020). In addition, workloads at home increased, with women bearing more of the burden. Much of that increased workload consisted of childcare and help with schooling, especially in remote areas with poor internet connectivity and no interaction with teachers (Cho et al. 2021). Survey data of Philippine households indicate that 40 per cent of women spent more time at home on at least three activities related to unpaid domestic work due to the pandemic, compared with 32 per cent of men (UN Women and Women Count 2020). This same survey also showed that women in the Philippines experienced larger declines than men in other sources of income, including remittances, savings and family businesses. Taking into account employment losses sustained abroad by migrant workers from the Philippines, who are predominantly women, the economy as a whole saw as much as a 14–20 per cent reduction in remittance inflows (Murakami, Shimizutani and Yamada 2021).

The pandemic is exposing some of the fault lines in the Philippines’ pre-pandemic gender equality situation, which will shape the country’s medium- and long-term recovery. Social norms on women’s reproductive roles affect women’s ability to actively engage in the labour market and are one factor explaining why the Philippines had the lowest female labour force participation rate in the Asia-Pacific region prior to the onset of the pandemic. According to analysis conducted for the National Economic and Development Authority (NEDA), the female labour force participation rate in the Philippines (46 per cent) was the lowest among all ASEAN countries; moreover, married women were 12 percentage points less likely to be employed than unmarried women, with larger gaps for women with young children (Cabegin and Gaddi 2019). Traditional gender roles and social expectations of women’s care responsibilities on the one hand, and men’s breadwinner responsibilities on the other, lead to more women leaving the labour market after marriage or childbirth.

Other areas of women’s sexual and reproductive health are associated with greater risk of exacerbating gender gaps. Before the COVID-19 pandemic, the

Philippines already had one of the highest rates of adolescent pregnancy (5 per cent) among Asian countries, and half of unmarried women of child-bearing age reported having an unmet need for family planning (PSA and ICF 2018). Estimates suggest that there were an additional 751,000 unintended pregnancies in 2020 as a result of reduced access to sexual and reproductive health services (Marquez et al. 2020). Increased birth rates in the Philippines during the pandemic will also contribute to more reproductive labour for women (Johnson, Simonette and Drury 2020). In the context of the COVID-19 pandemic and the ensuing economic downturn, it can be expected that these interlinking trends in the labour market, social norms and sexual and reproductive health will worsen, with long-term implications for women's economic empowerment and gender equality.

## 3. Simulation model

Our analysis considers the gendered distribution of the effects of the pandemic and lockdown restrictions by simulating how the demand and supply shocks associated with the pandemic translate into employment losses and changes in women's versus men's incomes. In so doing, our model accounts for both losses caused by the restrictions and reductions that would happen anyway because of fear of the virus.[5] Consistent with del Rio-Chanona et al. (2020), our simulation approach estimates the effects of first-order demand and supply shocks that reflect the direct and immediate impacts of containment policies and changes in behaviour to avoid exposure to the virus. We do not predict overall impacts that would include follow-up effects from further demand, and supply reductions and spillover from changes in the global supply chain.[6]

In this section, we model the shocks to employment through both demand and supply shocks. Next, we show how these shocks translate into a value-added reduction and then into income reduction. These reductions in turn are used to simulate the effect of the pandemic and lockdown on poverty.

### 3.1. Demand shocks

The presence of COVID-19 strongly influences patterns of consumer spending even in the absence of restrictions on business activity. In addition to increasing demand for health services, consumers are likely to try to reduce their risk of exposure to the virus and their demand for products and services that involve close contact with other people will decrease. To incorporate these behavioural patterns into our model, we follow the approach of del Rio-Chanona et al. (2020) and turn to estimates developed by the US Congressional Budget Office (CBO) that predict the potential impact of an influenza pandemic (United States, CBO 2006). The pandemic modelled by the CBO is based loosely on the Severe Acute Respiratory Syndrome Coronavirus (SARS-CoV-1) and reflects the behavioural responses that people would adopt to avoid infection in each industry. The CBO

[5] The analysis excludes the effects of fiscal policies, such as the payments in the initial stage of the pandemic under the Social Amelioration Program.

[6] Capturing these secondary effects would require a general equilibrium model, which could also build in scenarios such as the return of Filipino migrant workers.

analysis assumes that demand for goods and services entailing transmission risks, such as entertainment and travel, is reduced due to consumers' desire to avoid infection. The CBO provides two scenarios: one severe, one mild, where the demand shocks are predicted by industry based on expert opinions. The assumption underpinning the use of the CBO estimates is that while the structure of the economy of the Philippines differs from that of the United States in terms of sectoral weight, the behavioural responses of consumers in terms of changes to demand for specific products and services are likely to be similar. To perform this analysis, the CBO estimates used in del Rio-Chanona et al. (2020) are mapped to the Philippine Standard Industrial Classification (PSIC) industries.

The analysis assumes that consumers initially react to the presence of COVID-19 in a manner consistent with the severe shock scenario. As the public becomes desensitized to the presence of COVID-19 and the wave passes, it is assumed that shocks progress from severe to mild levels from mid-March 2020 to the end of that year.

## 3.2. Supply shocks

Restrictions on labour supply are present to varying degrees in each industry, region and period. These restrictions arise from social distancing requirements and closures of risky sectors as part of containment policies. Social distancing requirements are imposed during the periods of community quarantine according to the shares of workers permitted on site at a given time under Interagency Task Force announcements. Workers who cannot be on site do not necessarily become unproductive if they can work from home, but this depends on the home circumstances of employees (for example, if they have an adequate workspace or access to a computer) and the employer's willingness/ability to use distance arrangements. To reflect this, in addition to those workers permitted on site, 50 per cent of workers with occupations that would allow them to work from home are assumed to do so. The relative reductions modelled are based on which community quarantine lockdown level applies in each region and over each period. Each region is considered to have labour reductions per industry associated with the community quarantine policy in place for the proportion of the 2020 period analysed. Since community quarantine policies principally restrict the numbers of workers permitted on site, the policies are modelled as labour supply shocks.

As a developing country, much of the Philippines' economic output is generated by own-account production and informal employment. To capture effects via these channels, consequences for value added, $Y$, need to be modelled to reflect reduced demand for labour, $L$. The effects of supply shocks are modelled via a production function approach, where the shock is considered to be a shock to labour input. The proportional output of an industry $i$ in region $j$ in a period after the shock is taken as the share of remaining labour, raised to the power $\alpha$ (an output elasticity), which is approximated as the labour share of the industry's output in the region, as follows:

$$\left(\frac{Y_{ij} - \Delta Y_{ij}}{Y_{ij}}\right) = \left(1 - \frac{\Delta L_{ij}}{L_{ij}}\right)^{\alpha_{ij}} \tag{1}$$

The labour share is approximated as the total remuneration of workers in the industry (labour cost plus benefits and taxes), divided by the value of industry output, net of value-added taxes. The model is estimated using microdata from the Philippines' LFS and the FIES, and sample weights are used to calculate total remuneration by industry. The reduction in value added due to COVID-19 is taken as the proportional reduction multiplied by value added from the industry and region in 2019, prorated to the period's share of the year.

In the National Accounts, value added is published at the PSIC two-digit industry level nationally and at the one-digit industry level by region. LFS and FIES microdata enable calculation of the labour distribution within one-digit industry codes at the two-digit level by region. To obtain value added by region at the PSIC two-digit industry level, value added within each one-digit industry is assumed to follow the distribution of labour in the two-digit industry in each region multiplied by national average output for workers in that industry.

## 3.3. Net shocks

If value-added losses due to supply shocks are taken as the losses observed after restrictions on workers and are combined with losses from demand reductions, losses will be overestimated, given that reductions in demand may have led to a reduction in output in the industry, region and period anyway. For example, if tourism activities are not allowed and there is no demand for them, the reduction in tourism value added that would be observed is 100, not 200 per cent. To prevent this type of double counting, for a given industry, region and period, the larger of either the supply or demand shocks is taken as the reduction in value added. We note that this is conservative in terms of calculating losses, as effects on supply and demand are considered as substitutes, rather than as combined. However, actual losses may be additive, rather than substitutive. These changes in each period and region are prorated to the period's share of the year and then summed to give annual changes by region, which are in turn added together to give national changes.

## 3.4. Employment and income analysis

Our goal is to understand how the costs of lockdown measures are distributed among income groups and by gender. To do so, income shocks need to be simulated. In the case of wage income, this process is straightforward – reductions in labour demand equate to reductions in income, assuming that most employees can be freely dismissed or have their hours adjusted, and that unemployment benefits are not widely available. In the case of own-account workers or business owners, reductions in value added equate to reductions in income. To account for this type of production, the share of workers in an industry and region that are employed by others is multiplied by the labour reduction, while the share of workers who are not employed by others is multiplied by the value-added reduction. These values are then summed to obtain the supply-side proportional change in income.

On the demand side, reductions in output demand are translated into reductions in labour demand via a Cobb–Douglas production function approach as follows:

$$\frac{\Delta L_{ij}}{L_{ij}} = \left(1 - \frac{\delta_{ij}}{Y_{ij}}\right)^{\frac{1}{\alpha_{ij}}} \qquad (2)$$

In this case, the proportion of continuing output is raised to the inverse of the labour share, and the term $\delta/Y$ represents proportional value-added reduction. The greater of either the demand- or the supply-side-induced proportional reduction in income is taken for each period, industry and region. Reductions in each period are then prorated to the period's share of the year and added together to give annual proportional changes.

To translate the proportional changes into absolute changes in employment, the proportional changes are multiplied by the numbers of male and female workers in each PSIC code and region and then summed.

For income, the proportional changes are multiplied by weighted income reported for each industry in the region for households in the 2015 FIES microdata. This income is taken away from workers in uniform proportion across all workers in the industry and region, as it is not possible to determine which workers within the industry and region would be most likely to lose their jobs. This exercise is repeated for female and male incomes for each industry in each region, to capture the gender-differentiated effects of the industry and regional restrictions, based on the share of employment and income by gender in each industry and region.

To calculate effects on poverty, income after adjustments is aggregated at the household level using FIES 2015 microdata and weighted to reflect provincial and national household populations. Then, numbers of households falling below 2015 provincial poverty lines, as well as other poverty measures, are counted and compared with actual 2015 values using a range of groupings to explore effects on different subpopulations of interest.

## 4. Results

Results from estimating equation (1) indicate substantial reductions in economic activity, with a decline of 13.5 per cent nationally (table 1). Losses of economic activity are concentrated in both more urban regions subjected to longer and more intensive lockdown measures, and regions dependent on heavily affected sectors, such as tourism. We note that this modelled reduction in economic activity is similar to the actual decline in GDP growth, which fell by 15.5 percentage points, from 6.0 per cent in 2019 to –9.5 per cent in 2020. Given that the decline in the model is slightly lower than actual values, the results presented may slightly underestimate effects.

Simulated effects on labour demand and employment are even stronger than effects on value added and differ substantially by gender. Our modelling points to a 20 per cent larger reduction in labour for women compared with men, reflecting the higher shares of female workers in some of the industries that have faced the greatest losses, such as travel agencies and employment-related activities (table 2). Overall, women experience a 27.0 per cent decline in employment, compared with 22.5 per cent for men. Higher relative reductions in female labour demand are found for every region of the Philippines.

### Table 1. Simulated reductions in economic activity in the Philippines resulting from the COVID-19 pandemic and related policy responses by region (millions of Philippine pesos)

| Region | Baseline, 2019 | Value-added loss, 2020 | Value-added loss, 2020 (%) |
|---|---|---|---|
| Region I (Ilocos Region) | 650 542 | 95 187 | 14.6 |
| Region II (Cagayan Valley) | 396 522 | 52 317 | 13.2 |
| Region III (Central Luzon) | 2 173 084 | 305 165 | 14.0 |
| Region IV-A (CALABARZON) | 2 855 689 | 400 795 | 14.0 |
| MIMAROPA Region | 376 103 | 54 043 | 14.4 |
| Region V (Bicol Region) | 556 981 | 78 335 | 14.1 |
| Region VI (Western Visayas) | 912 135 | 142 501 | 15.6 |
| Region VII (Central Visayas) | 1 262 484 | 239 350 | 19.0 |
| Region VIII (Eastern Visayas) | 463 608 | 46 392 | 10.0 |
| Region IX (Zamboanga Peninsula) | 395 042 | 42 810 | 10.8 |
| Region X (Northern Mindanao) | 877 080 | 75 570 | 8.6 |
| Region XI (Davao Region) | 919 092 | 114 284 | 12.4 |
| Region XII (SOCCSKSARGEN) | 467 502 | 49 821 | 10.7 |
| National Capital Region (NCR) | 6 235 408 | 816 224 | 13.1 |
| Cordillera Administrative Region (CAR) | 320 695 | 42 926 | 13.4 |
| Autonomous Region in Muslim Mindanao (ARMM) | 244 840 | 24 960 | 10.2 |
| Region XIII (Caraga) | 304 341 | 32 918 | 10.8 |
| **Total** | **19 411 148** | **2 613 598** | **13.5** |

Notes: Region names as per the Philippine Standard Geographic Codes (Philippine Statistics Authority) as of 30 June 2021. Average exchange rate for 2019–20, US$1 = PHP50.71 (Bangko Sentral ng Pilipinas).

Source: Authors' estimates.

### Table 2. Simulated reductions in labour demand in the Philippines resulting from the COVID-19 pandemic and related policy responses by region

| Region | Baseline employment, 2019 | | Loss of labour demand, 2020 | | Employment reduction, 2020 (%) | |
|---|---|---|---|---|---|---|
| | Men | Women | Men | Women | Men | Women |
| Region I (Ilocos Region) | 1 306 665 | 778 096 | 279 273 | 200 453 | 21.4 | 25.8 |
| Region II (Cagayan Valley) | 957 307 | 551 377 | 155 408 | 121 503 | 16.2 | 22.0 |
| Region III (Central Luzon) | 2 634 589 | 1 654 403 | 723 220 | 518 487 | 27.5 | 31.3 |
| Region IV-A (CALABARZON) | 2 985 655 | 2 243 622 | 857 668 | 717 266 | 28.7 | 32.0 |
| MIMAROPA Region | 806 309 | 496 966 | 141 971 | 118 502 | 17.6 | 23.8 |
| Region V (Bicol Region) | 1 473 980 | 901 111 | 279 026 | 230 561 | 18.9 | 25.6 |
| Region VI (Western Visayas) | 1 971 874 | 1 324 941 | 382 150 | 330 436 | 19.4 | 24.9 |
| Region VII (Central Visayas) | 1 781 313 | 1 364 460 | 492 640 | 420 389 | 27.7 | 30.8 |
| Region VIII (Eastern Visayas) | 1 129 613 | 683 429 | 173 071 | 135 737 | 15.3 | 19.9 |
| Region IX (Zamboanga Peninsula) | 890 688 | 524 300 | 140 064 | 103 945 | 15.7 | 19.8 |
| Region X (Northern Mindanao) | 1 182 715 | 744 754 | 208 621 | 143 857 | 17.6 | 19.3 |
| Region XI (Davao Region) | 1 229 454 | 716 435 | 257 032 | 179 461 | 20.9 | 25.0 |
| Region XII (SOCCSKSARGEN) | 1 076 855 | 658 210 | 167 725 | 132 760 | 15.6 | 20.2 |
| National Capital Region (NCR) | 2 709 564 | 2 223 586 | 887 634 | 742 440 | 32.8 | 33.4 |
| Cordillera Administrative Region (CAR) | 458 424 | 306 969 | 80 021 | 65 878 | 17.5 | 21.5 |
| Autonomous Region in Muslim Mindanao (ARMM) | 889 397 | 329 524 | 100 601 | 49 995 | 11.3 | 15.2 |
| Region XIII (Caraga) | 669 929 | 395 233 | 110 759 | 82 801 | 16.5 | 21.0 |
| **Total** | **24 154 330** | **15 897 418** | **5 436 882** | **4 294 471** | **22.5** | **27.0** |

Note: Region names as per the Philippine Standard Geographic Codes (Philippine Statistics Authority) as of 30 June 2021.

Source: Authors' estimates.

Simulation results also indicate that the reductions in labour demand translates into substantial effects on 2020 household income, with losses of 29.1 per cent of income generated by female workers and 26.2 per cent of income generated by male workers (table 3), which are both much higher than the 13.5 per cent reduction in national value added modelled. Hence the relative reduction for women is more than 10 per cent larger than that for men. The national pattern is mirrored in all regions except the National Capital Region, where relative reductions are nearly equal between men and women. In absolute terms, losses for men are greater than those for women, largely due to higher wages and labour force participation among men prior to the COVID-19 pandemic.

Many of these income losses hit the poor and vulnerable hardest. Poverty is modelled to nearly double in the Philippines relative to the baseline without COVID-19, with a 73.3 per cent increase nationally under containment policies adopted in 2020 (table 4). This increase in poverty is more pronounced in urban than in rural areas, unlike many other economic shocks encountered in the past. Part of this disproportionate impact in urban areas is due to the industries affected by demand changes driven by the presence of the pandemic, but the main explanation is the concentration of lockdown measures in more urban areas. Female-headed households are much more adversely affected than male-headed households, but this disparity arises primarily in rural rather than in

**Table 3. Simulated reductions in household income in the Philippines resulting from COVID-19 and related policy responses by region (millions of Philippine pesos)**

| Region | Baseline income, 2019 | | Income loss, 2020 | | Income loss, 2020 (%) | |
|---|---|---|---|---|---|---|
| | Men | Women | Men | Women | Men | Women |
| Region I (Ilocos Region) | 105 071 | 58 869 | 25 905 | 15 910 | 24.7 | 27.0 |
| Region II (Cagayan Valley) | 69 992 | 38 232 | 14 297 | 10 510 | 20.4 | 27.5 |
| Region III (Central Luzon) | 284 726 | 170 700 | 85 040 | 55 117 | 29.9 | 32.3 |
| Region IV-A (CALABARZON) | 348 954 | 235 167 | 103 655 | 72 841 | 29.7 | 31.0 |
| MIMAROPA Region | 55 603 | 33 664 | 11 345 | 9 683 | 20.4 | 28.8 |
| Region V (Bicol Region) | 104 211 | 66 988 | 23 238 | 19 256 | 22.3 | 28.7 |
| Region VI (Western Visayas) | 139 986 | 100 753 | 31 413 | 27 073 | 22.4 | 26.9 |
| Region VII (Central Visayas) | 154 220 | 109 405 | 49 135 | 37 607 | 31.9 | 34.4 |
| Region VIII (Eastern Visayas) | 80 575 | 53 618 | 14 350 | 11 725 | 17.8 | 21.9 |
| Region IX (Zamboanga Peninsula) | 63 450 | 36 863 | 12 118 | 9 087 | 19.1 | 24.7 |
| Region X (Northern Mindanao) | 96 046 | 56 769 | 18 847 | 12 671 | 19.6 | 22.3 |
| Region XI (Davao Region) | 108 506 | 67 385 | 24 682 | 17 321 | 22.7 | 25.7 |
| Region XII (SOCCSKSARGEN) | 71 579 | 47 866 | 12 894 | 10 964 | 18.0 | 22.9 |
| National Capital Region (NCR) | 447 074 | 324 022 | 143 146 | 101 761 | 32.0 | 31.4 |
| Cordillera Administrative Region (CAR) | 41 539 | 28 345 | 9 189 | 7 386 | 22.1 | 26.1 |
| Autonomous Region in Muslim Mindanao (ARMM) | 49 254 | 18 093 | 6 643 | 4 153 | 13.5 | 23.0 |
| Region XIII (Caraga) | 54 582 | 34 587 | 10 691 | 8 209 | 19.6 | 23.7 |
| **Total** | **2 275 366** | **1 481 327** | **596 589** | **431 274** | **26.2** | **29.1** |

Notes: Region names as per the Philippine Standard Geographic Codes (Philippine Statistics Authority) as of 30 June 2021. Average exchange rate for 2019–20, US$1 = PHP50.71 (Bangko Sentral ng Pilipinas).

Source: Authors' estimates.

**Table 4. Simulated changes in poverty in the Philippines resulting from the COVID-19 pandemic and related policy responses by household type**

| Household type | | Relative poverty rate (%) | Income inequality | |
|---|---|---|---|---|
| | | | Gini | P75/P25 |
| All | Pandemic only | 140.4 | 103.5 | 102.5 |
| | Containment policies | 173.3 | 100.1 | 94.6 |
| Urban | Pandemic only | 174.9 | 105.3 | 108.4 |
| | Containment policies | 237.2 | 103.9 | 102.5 |
| Rural | Pandemic only | 131.2 | 103.1 | 101.0 |
| | Containment policies | 156.2 | 98.3 | 96.0 |
| Female headed | Pandemic only | 147.4 | 102.0 | 102.6 |
| | Containment policies | 190.4 | 102.1 | 99.9 |
| Male headed | Pandemic only | 139.3 | 102.5 | 102.0 |
| | Containment policies | 170.7 | 97.9 | 94.3 |
| Female headed – urban | Pandemic only | 168.7 | 105.2 | 106.6 |
| | Containment policies | 232.0 | 106.9 | 105.4 |
| Male headed – urban | Pandemic only | 176.2 | 104.6 | 109.8 |
| | Containment policies | 238.3 | 101.8 | 102.5 |
| Female headed – rural | Pandemic only | 138.0 | 102.0 | 101.0 |
| | Containment policies | 171.9 | 100.5 | 96.8 |
| Male headed – rural | Pandemic only | 130.3 | 103.6 | 101.0 |
| | Containment policies | 154.1 | 97.7 | 95.7 |

Note: The P75/P25 measure is the ratio of workers' wages in the 75th and 25th percentiles.
Source: Authors' estimates.

urban areas. Even in the absence of containment policies, demand shocks affect female-headed household poverty more than that of male-headed households, but containment policies widen the disparity.

## 5. Case study: Short-term costs to parents and teachers

School closures have additional labour market consequences, in terms of losses both in the employment of private school teachers and in income for parents who have to provide childcare. Private school enrolment fell by 48 per cent in the 2020/21 school year, according to Department of Education enrolment figures from September 2020 (Luz 2020). According to the Coordinating Council of Private Educational Associations, private schools in the Philippines employ 409,757 teachers and other staff (Magsambol 2020). Data from the LFS indicate that women represent two thirds of all teachers. Based on merged FIES and LFS data, we assume that 30 per cent of those employees lose their jobs. This change amounts to approximately 123,000 jobs lost in the 2020/21 school year, with an estimated wage loss of 16 billion Philippine pesos (PHP).

As for the losses in parents' incomes, we used the merged 2015 FIES and LFS data to identify all households with children below the age of 12 whose parents are both working. We assume that an adult needs to have at least a high-school education in order to support children's online learning. We also assume that in households where both parents are working and where there are no grandparents under the age of 80 with at least a high-school education, one parent would need to stay at home and this parent would be the one with lower earnings. In such households, approximately 92 per cent of fathers earn more than mothers. Mothers have an average schooling of 9.5 years compared with 8.5 years for fathers. A combination of lower earnings and longer schooling makes mothers the parent who is most likely to stop working to support children's online learning. About one third of households in the data set meet these criteria. Our simulation shows a projected overall loss of 6.0 per cent of household income, or PHP225 billion for one school year. Most of this loss (PHP207 billion) reflects women's lost wages as a result of staying home to provide childcare and home schooling, whereby female income declines by 14 per cent, versus male income, which declines by 0.1 per cent. There are additional costs in terms of lost wages of private school teachers due to school closures, at PHP10.7 billion for female teachers and PHP5.3 billion for male teachers.

In the medium-to-long term, protracted face-to-face school closures threaten the ability of the country's youth to be productive as workers, and this threat falls most heavily on the poorest households, which struggle the most to support distance learning arrangements. A higher percentage of children have stopped going to school in the Philippines than in other countries in the region, just as a relatively low percentage of children (21 per cent) have been attending online classes (if they have been offered) due to weak internet connections and a lack of digital devices (Morgan and Trinh 2021). Judging by the higher school dropout rates for girls in the Philippines during the Asian financial crisis of 1998–99, girls may not return to school as quickly as boys following the COVID-19 school closures (World Bank 2020). Women's job losses and higher school dropout rates for girls, combined with the increases in unintended pregnancies reported in the media, will not only roll back gender equality gains but will also risk limiting economic recovery efforts by reducing skilled work opportunities for a significant share of the population.

## 6. Conclusion and policy implications

The COVID-19 pandemic and the associated containment policies in the Philippines have contributed to enormous constraints on the ability of workers to engage in employment and contribute to the economy, affecting women relatively more than men. The pandemic has shone a spotlight on the vulnerability of the poor, and poor women in particular, to economic crisis, and our results show that low-income individuals are bearing relatively more of the costs in the Philippines. The pandemic is exposing the fault lines of the existing system (social norms, services, infrastructure) and, in particular, the disparities in the lived experiences of men and women, and of different income groups. Our simulation analysis shows substantial short-run increases in poverty that may take years to overcome. These poverty effects will in turn affect recovery strategies.

At the time of writing, the lockdown policies generating many of these losses were planned to continue into 2021. However, the losses observed in this article need not continue. There are alternatives to broad lockdowns and blanket school closures that can achieve even greater control of the pandemic without sacrificing the future welfare of women and the poor. For example, Chen et al. (2020) find large effects from targeted contact-reducing measures, contact tracing and paid sick leave, and somewhat smaller effects of school closures and movement restrictions. More reliance on such approaches can help to reduce the losses facing disadvantaged groups.

Given the adverse direct and indirect consequences of the pandemic and the lockdown measures on gender equality in the Philippines, recovery strategies should take into account how traditional social norms and gender inequalities interlock, in order to promote a more equitable recovery. Even though the Philippines has made great progress on gender equality in recent decades, expectations that women are to fulfil traditional reproductive roles are an important factor in low female labour force participation and women's particular vulnerability to poverty in the ongoing pandemic. The Government of the Philippines has been proactive in terms of providing health insurance coverage for COVID-19, as well as cash transfers and food subsidies for poor households (Park and Inocencio 2020). Because COVID-19 risks unravelling the progress made on gender equality, the Government's policy response strategy needs to place even greater emphasis on a care-led recovery with generous social protection, countercyclical measures to boost aggregate demand, and investment in vital care systems. Public investment in both childcare and long-term care services can create millions of jobs, both directly in the care sector, and indirectly through induced economic activity in some of the hardest-hit industries, such as retail, healthcare and food services, which disproportionately employ women. The effects of the lockdown measures adopted thus far will require mitigation, such as through comprehensive support to enable women to access decent work and skills upgrading opportunities, including expansion of good-quality and affordable childcare provision. Also crucial are dedicated efforts to bring girls back to school, given their relatively higher probability of dropping out.

Ensuring that reproductive and sexual health services are available to women and girls as part of the pandemic response and recovery strategy is similarly vital in bringing down the high number of unintended pregnancies and early marriages, and in protecting maternal and infant health. Adolescent and unintended pregnancies are not only linked with poorer health outcomes for women and their children but they are also likely to increase women's vulnerability to poverty and to reduce their opportunities to enter the labour market or continue their education. Recognizing the importance of tackling the problem of teen pregnancies during the pandemic, the Commission on Population of the Philippines has launched new initiatives aimed at mobilizing and raising awareness among local government units, health providers, and communities. Scaling up such initiatives will be critical. Ring-fencing public budgets, expanding telehealth services and exploring additional avenues for increasing access to family planning during lockdowns will all help to safeguard the health and well-being of women and their children, thus helping to counteract the negative effects of the lockdowns on women's employment and income that are documented in our study.
